# 2,2,2-Trichloro-*N*-(2,5-dimethyl­phen­yl)acetamide

**DOI:** 10.1107/S1600536808009264

**Published:** 2008-04-10

**Authors:** B. Thimme Gowda, Sabine Foro, Hartmut Fuess

**Affiliations:** a Department of Chemistry, Mangalore University, Mangalagangotri 574 199, Mangalore, India; b Institute of Materials Science, Darmstadt University of Technology, Petersenstrasse 23, D-64287 Darmstadt, Germany

## Abstract

The N—H bond in the title compound, C_10_H_10_Cl_3_NO, is *syn* to the 2-methyl and *anti* to the 5-methyl substituent of the aromatic ring. Adjacent mol­ecules are linked into chains through N—H⋯O hydrogen bonding. Two Cl atoms are each disordered equally over two sites.

## Related literature

For related literature, see: Gowda, Foro & Fuess (2007 ▶); Gowda, Kožíšek *et al*. (2007 ▶); Shilpa & Gowda (2007 ▶).
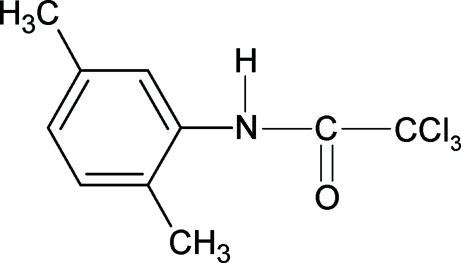


## Experimental

### 

#### Crystal data

C_10_H_10_Cl_3_NO
*M* *_r_* = 266.54
Orthorhombic, 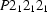

*a* = 4.9173 (9) Å
*b* = 11.290 (1) Å
*c* = 21.070 (2) Å
*V* = 1169.7 (3) Å^3^
*Z* = 4
Mo *K*α radiation
μ = 0.76 mm^−1^
*T* = 299 (2) K
0.16 × 0.12 × 0.06 mm

#### Data collection

Oxford Diffraction Xcalibur diffractometer with Sapphire CCD detector
Absorption correction: multi-scan (*CrysAlis RED*; Oxford Diffraction, 2007 ▶) *T*_min_ = 0.889, *T*_max_ = 0.956
6121 measured reflections
2314 independent reflections
703 reflections with *I* > 2σ(*I*)
*R*_int_ = 0.071

#### Refinement

*R*[*F* ^2^ > 2σ(*F* ^2^)] = 0.088
*wR*(*F* ^2^) = 0.308
*S* = 0.86
2314 reflections
154 parameters
37 restraints
H-atom parameters constrained
Δρ_max_ = 0.27 e Å^−3^
Δρ_min_ = −0.76 e Å^−3^
Absolute structure: Flack (1983 ▶), 887 Friedel pairs
Flack parameter: −0.4 (4)

### 

Data collection: *CrysAlis CCD* (Oxford Diffraction, 2004 ▶); cell refinement: *CrysAlis RED* (Oxford Diffraction, 2007 ▶); data reduction: *CrysAlis RED*; program(s) used to solve structure: *SHELXS97* (Sheldrick, 2008 ▶); program(s) used to refine structure: *SHELXL97* (Sheldrick, 2008 ▶); molecular graphics: *PLATON* (Spek, 2003 ▶); software used to prepare material for publication: *SHELXL97*.

## Supplementary Material

Crystal structure: contains datablocks I, global. DOI: 10.1107/S1600536808009264/ng2441sup1.cif

Structure factors: contains datablocks I. DOI: 10.1107/S1600536808009264/ng2441Isup2.hkl

Additional supplementary materials:  crystallographic information; 3D view; checkCIF report

## Figures and Tables

**Table 1:** Hydrogen-bond geometry (Å, °)

*D*—H⋯*A*	*D*—H	H⋯*A*	*D*⋯*A*	*D*—H⋯*A*
N7—H7N⋯O6^i^	0.86	2.12	2.984 (11)	178

Symmetry code: (i) 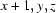.

**Fig. 1.**
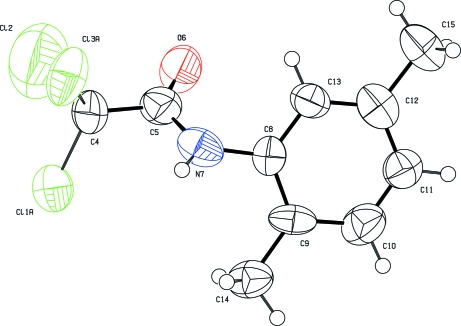
Molecular structure of the title compound, showing the atom labeling scheme. The displacement ellipsoids drawn at the 50% probability level.

**Fig. 2.**
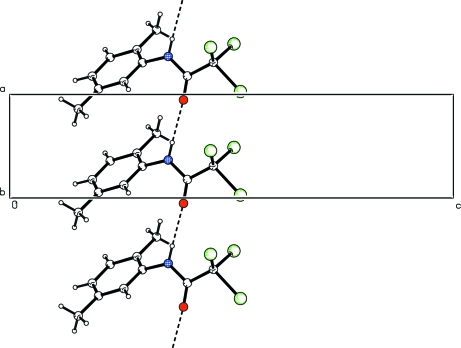
Molecular packing of the title compound with hydrogen bonding shown as dashed lines.

## supplementary crystallographic information

### Comment

In the present work, the structure of 2,2,2-trichloro-*N*- (2,5-dimethylphenyl)acetamide (25DMPTCA) has been determined to study the effect of substituents on the structures of *N*-aromatic amides (Gowda, Foro *et al.*, 2007; Gowda, Kožíšek *et al.*, 2007). The conformation of the N—H bond in 25DMPTCA is *syn* to the 2-methyl and *anti* to the 5-methyl substituents in the aromatic ring (Fig. 1), similar to the *syn* conformation observed with respect to the 2-methyl substituent in 2,2,2-trichloro-*N*-(2-methylphenyl)acetamide (2MPTCA) (Gowda, Kožíšek *et al.*, 2007). The bond parameters in 25DMPTCA are similar to those in 2MPTCA, 2,2,2-trichloro-*N*-(2,6-dimethylphenyl)- acetamide and other acetanilides (Gowda, Foro *et al.*, 2007; Gowda, Kožíšek *et al.*, 2007). The intermolecular N—H···O hydrogen bonds link the molecules into chains (Table 1 and Fig.2). The Cl atoms of CCl_3_ group are disordered and Cl1 and Cl3 were refined using a split model with site-occupation factors 0.5:0.5. No reliable disorder model could be produced for Cl2.

### Experimental

The title compound was prepared according to the literature method (Shilpa and Gowda, 2007). The purity of the compound was checked by determining its melting point. It was characterized by recording its infrared and NMR spectra (Shilpa and Gowda, 2007). Single crystals of the title compound were obtained from an ethanolic solution and used for X-ray diffraction studies at room temperature.

### Refinement

The H atoms were positioned with idealized geometry using a riding model with C—H = 0.93–0.96 Å, N—H = 0.86 Å. All H atoms were refined with isotropic displacement parameters (set to 1.2 times of the *U*_eq_ of the parent atom).

The Cl atoms of CCl_3_ group are disordered and Cl1 and Cl3 were refined using a split model with site-occupation factors 0.5:0.5. No reliable disorder model could be produced for Cl2. The C—Cl distances were restrained to 1.77 (2) Å and the distances in the disordered groups were restrained to be equal.

The compound is a weak anamalous scatterer with minor intensity at high θ value. The low fraction of unique data is above the 2σ level (30°).

### Crystal data

**Table:**

C_10_H_10_Cl_3_NO	*F*_000_ = 544
*M**_r_* = 266.54	*D*_x_ = 1.514 Mg m^−^^3^
Orthorhombic, *P*2_1_2_1_2_1_	Mo *K*α radiation λ = 0.71073 Å
Hall symbol: P 2ac 2ab	Cell parameters from 1061 reflections
*a* = 4.9173 (9) Å	θ = 2.6–28.1º
*b* = 11.290 (1) Å	µ = 0.76 mm^−^^1^
*c* = 21.070 (2) Å	*T* = 299 (2) K
*V* = 1169.7 (3) Å^3^	Prism, colourless
*Z* = 4	0.16 × 0.12 × 0.06 mm

### Data collection

**Table:**

Oxford Diffraction Xcalibur diffractometer with Sapphire CCD detector	2314 independent reflections
Radiation source: Enhance (Mo) X-ray Source	703 reflections with *I* > 2σ(*I*)
Monochromator: graphite	*R*_int_ = 0.071
*T* = 299(2) K	θ_max_ = 26.4º
Rotation method data acquisition using ω and phi scans.	θ_min_ = 3.4º
Absorption correction: multi-scan(CrysAlis RED; Oxford Diffraction, 2007)	*h* = −6→6
*T*_min_ = 0.889, *T*_max_ = 0.956	*k* = −14→13
6121 measured reflections	*l* = −26→23

### Refinement

**Table:**

Refinement on *F*^2^	Hydrogen site location: inferred from neighbouring sites
Least-squares matrix: full	H-atom parameters constrained
*R*[*F*^2^ > 2σ(*F*^2^)] = 0.088	*w* = 1/[σ^2^(*F*_o_^2^) + (0.1675*P*)^2^] where *P* = (*F*_o_^2^ + 2*F*_c_^2^)/3
*wR*(*F*^2^) = 0.309	(Δ/σ)_max_ = 0.003
*S* = 0.86	Δρ_max_ = 0.27 e Å^−^^3^
2314 reflections	Δρ_min_ = −0.76 e Å^−^^3^
154 parameters	Extinction correction: none
37 restraints	Absolute structure: Flack (1983), 887 Friedel pairs
Primary atom site location: structure-invariant direct methods	Flack parameter: −0.4 (4)
Secondary atom site location: difference Fourier map	

### Special details

**Table:**

Experimental. empirical absorption correction using spherical harmonics, implemented in SCALE3 ABSPACK scaling algorithm
Geometry. All e.s.d.'s (except the e.s.d. in the dihedral angle between two l.s. planes) are estimated using the full covariance matrix. The cell e.s.d.'s are taken into account individually in the estimation of e.s.d.'s in distances, angles and torsion angles; correlations between e.s.d.'s in cell parameters are only used when they are defined by crystal symmetry. An approximate (isotropic) treatment of cell e.s.d.'s is used for estimating e.s.d.'s involving l.s. planes.
Refinement. Refinement of *F*^2^ against ALL reflections. The weighted *R*-factor *wR* and goodness of fit *S* are based on *F*^2^, conventional *R*-factors *R* are based on *F*, with *F* set to zero for negative *F*^2^. The threshold expression of *F*^2^ > σ(*F*^2^) is used only for calculating *R*-factors(gt) *etc*. and is not relevant to the choice of reflections for refinement. *R*-factors based on *F*^2^ are statistically about twice as large as those based on *F*, and *R*- factors based on ALL data will be even larger.

### Fractional atomic coordinates and isotropic or equivalent isotropic displacement parameters (Å^2^)

**Table:**

	*x*	*y*	*z*	*U*_iso_*/*U*_eq_	Occ. (<1)
Cl1A	0.4909 (19)	0.3895 (7)	0.5055 (3)	0.096 (2)	0.50
Cl1B	0.301 (3)	0.3888 (10)	0.5125 (4)	0.139 (4)	0.50
Cl2	0.0276 (14)	0.5253 (7)	0.5201 (3)	0.218 (3)	
Cl3A	0.459 (2)	0.6280 (6)	0.4526 (3)	0.102 (2)	0.50
Cl3B	0.226 (2)	0.6324 (7)	0.4737 (5)	0.141 (3)	0.50
O6	−0.0518 (17)	0.4448 (7)	0.3920 (4)	0.092 (3)	
N7	0.3670 (16)	0.4054 (7)	0.3571 (4)	0.069 (2)	
H7N	0.5356	0.4163	0.3662	0.083*	
C4	0.311 (2)	0.4931 (7)	0.4592 (4)	0.082 (3)	
C5	0.180 (3)	0.4444 (9)	0.4003 (6)	0.075 (3)	
C8	0.308 (2)	0.3488 (8)	0.2991 (4)	0.055 (3)	
C9	0.4334 (19)	0.2416 (9)	0.2866 (5)	0.062 (3)	
C10	0.358 (2)	0.1877 (10)	0.2267 (5)	0.079 (3)	
H10	0.4336	0.1151	0.2154	0.095*	
C11	0.178 (2)	0.2420 (10)	0.1863 (4)	0.067 (3)	
H11	0.1371	0.2054	0.1480	0.080*	
C12	0.055 (2)	0.3486 (10)	0.2002 (5)	0.069 (3)	
C13	0.125 (2)	0.4011 (9)	0.2572 (4)	0.062 (3)	
H13	0.0466	0.4735	0.2679	0.074*	
C14	0.624 (2)	0.1827 (8)	0.3309 (5)	0.075 (3)	
H14A	0.5312	0.1655	0.3700	0.090*	
H14B	0.7750	0.2341	0.3392	0.090*	
H14C	0.6878	0.1103	0.3123	0.090*	
C15	−0.138 (2)	0.4037 (11)	0.1548 (5)	0.091 (3)	
H15A	−0.2838	0.3497	0.1462	0.109*	
H15B	−0.0441	0.4218	0.1160	0.109*	
H15C	−0.2094	0.4753	0.1727	0.109*	

### Atomic displacement parameters (Å^2^)

**Table:**

	*U*^11^	*U*^22^	*U*^33^	*U*^12^	*U*^13^	*U*^23^
Cl1A	0.116 (6)	0.109 (5)	0.063 (3)	0.023 (5)	0.000 (4)	−0.003 (3)
Cl1B	0.172 (8)	0.151 (7)	0.093 (5)	−0.036 (7)	0.005 (6)	0.037 (5)
Cl2	0.213 (6)	0.278 (7)	0.163 (4)	0.011 (6)	0.007 (4)	−0.053 (4)
Cl3A	0.135 (6)	0.086 (4)	0.086 (4)	−0.038 (4)	0.016 (4)	−0.025 (3)
Cl3B	0.151 (7)	0.117 (6)	0.154 (6)	0.018 (6)	−0.027 (6)	−0.049 (5)
O6	0.052 (4)	0.127 (7)	0.099 (5)	0.011 (5)	−0.007 (4)	−0.040 (5)
N7	0.049 (5)	0.068 (5)	0.092 (6)	−0.004 (5)	−0.014 (5)	0.016 (5)
C4	0.091 (8)	0.070 (7)	0.084 (7)	0.002 (7)	−0.025 (7)	−0.019 (6)
C5	0.056 (7)	0.081 (8)	0.088 (7)	0.011 (7)	−0.018 (7)	−0.019 (6)
C8	0.055 (6)	0.061 (6)	0.048 (5)	−0.009 (6)	0.004 (6)	−0.005 (5)
C9	0.045 (5)	0.056 (6)	0.086 (7)	0.011 (6)	0.004 (6)	0.018 (6)
C10	0.079 (8)	0.064 (7)	0.095 (8)	−0.004 (7)	0.000 (7)	−0.012 (6)
C11	0.077 (8)	0.064 (7)	0.061 (6)	−0.004 (7)	0.005 (6)	0.005 (5)
C12	0.063 (7)	0.078 (8)	0.066 (6)	−0.016 (7)	−0.003 (6)	0.022 (6)
C13	0.062 (6)	0.062 (5)	0.062 (6)	0.006 (6)	−0.005 (6)	0.010 (5)
C14	0.073 (7)	0.050 (6)	0.102 (7)	0.001 (7)	−0.004 (7)	0.000 (6)
C15	0.080 (8)	0.119 (9)	0.074 (6)	−0.001 (9)	−0.017 (7)	0.019 (7)

### Geometric parameters (Å, °)

**Table:**

Cl1A—C4	1.761 (11)	C10—C11	1.373 (14)
Cl1B—C4	1.628 (11)	C10—H10	0.9300
Cl2—C4	1.931 (10)	C11—C12	1.378 (13)
Cl3A—C4	1.693 (10)	C11—H11	0.9300
Cl3B—C4	1.656 (10)	C12—C13	1.382 (14)
O6—C5	1.151 (11)	C12—C15	1.484 (14)
N7—C5	1.367 (13)	C13—H13	0.9300
N7—C8	1.411 (11)	C14—H14A	0.9600
N7—H7N	0.8600	C14—H14B	0.9600
C4—C5	1.503 (13)	C14—H14C	0.9600
C8—C9	1.383 (12)	C15—H15A	0.9600
C8—C13	1.393 (13)	C15—H15B	0.9600
C9—C10	1.449 (14)	C15—H15C	0.9600
C9—C14	1.480 (12)		
			
C5—N7—C8	125.7 (8)	C10—C9—C14	121.5 (9)
C5—N7—H7N	117.1	C11—C10—C9	121.1 (10)
C8—N7—H7N	117.1	C11—C10—H10	119.5
C5—C4—Cl1B	106.9 (8)	C9—C10—H10	119.5
C5—C4—Cl3B	113.0 (8)	C10—C11—C12	122.8 (10)
Cl1B—C4—Cl3B	123.5 (8)	C10—C11—H11	118.6
C5—C4—Cl3A	116.5 (7)	C12—C11—H11	118.6
Cl1B—C4—Cl3A	136.1 (7)	C11—C12—C13	116.8 (9)
Cl3B—C4—Cl3A	43.0 (5)	C11—C12—C15	120.6 (10)
C5—C4—Cl1A	115.4 (7)	C13—C12—C15	122.6 (11)
Cl1B—C4—Cl1A	32.1 (4)	C12—C13—C8	121.9 (9)
Cl3B—C4—Cl1A	131.0 (7)	C12—C13—H13	119.1
Cl3A—C4—Cl1A	115.4 (7)	C8—C13—H13	119.1
C5—C4—Cl2	107.8 (8)	C9—C14—H14A	109.5
Cl1B—C4—Cl2	69.8 (7)	C9—C14—H14B	109.5
Cl3B—C4—Cl2	61.1 (6)	H14A—C14—H14B	109.5
Cl3A—C4—Cl2	101.2 (5)	C9—C14—H14C	109.5
Cl1A—C4—Cl2	96.8 (6)	H14A—C14—H14C	109.5
O6—C5—N7	124.5 (10)	H14B—C14—H14C	109.5
O6—C5—C4	123.3 (12)	C12—C15—H15A	109.5
N7—C5—C4	112.1 (10)	C12—C15—H15B	109.5
C9—C8—C13	122.6 (9)	H15A—C15—H15B	109.5
C9—C8—N7	118.0 (9)	C12—C15—H15C	109.5
C13—C8—N7	119.4 (9)	H15A—C15—H15C	109.5
C8—C9—C10	114.8 (9)	H15B—C15—H15C	109.5
C8—C9—C14	123.7 (9)		
			
C8—N7—C5—O6	−7.0 (17)	C13—C8—C9—C10	−0.2 (13)
C8—N7—C5—C4	176.0 (8)	N7—C8—C9—C10	178.7 (9)
Cl1B—C4—C5—O6	84.0 (14)	C13—C8—C9—C14	−178.4 (9)
Cl3B—C4—C5—O6	−55.0 (16)	N7—C8—C9—C14	0.6 (13)
Cl3A—C4—C5—O6	−102.5 (14)	C8—C9—C10—C11	0.6 (14)
Cl1A—C4—C5—O6	117.4 (13)	C14—C9—C10—C11	178.8 (9)
Cl2—C4—C5—O6	10.4 (14)	C9—C10—C11—C12	−1.1 (17)
Cl1B—C4—C5—N7	−99.0 (10)	C10—C11—C12—C13	1.0 (15)
Cl3B—C4—C5—N7	121.9 (10)	C10—C11—C12—C15	179.7 (10)
Cl3A—C4—C5—N7	74.5 (11)	C11—C12—C13—C8	−0.6 (14)
Cl1A—C4—C5—N7	−65.6 (11)	C15—C12—C13—C8	−179.3 (9)
Cl2—C4—C5—N7	−172.6 (7)	C9—C8—C13—C12	0.3 (14)
C5—N7—C8—C9	−127.3 (10)	N7—C8—C13—C12	−178.7 (9)
C5—N7—C8—C13	51.7 (12)		

### Hydrogen-bond geometry (Å, °)

**Table:**

*D*—H···*A*	*D*—H	H···*A*	*D*···*A*	*D*—H···*A*
N7—H7N···O6^i^	0.86	2.12	2.984 (11)	178

Symmetry codes: (i) *x*+1, *y*, *z*.
